# Low Molecular Weight Gelators Based on Functionalized l-Dopa Promote Organogels Formation

**DOI:** 10.3390/gels5020027

**Published:** 2019-05-14

**Authors:** Demetra Giuri, Nicola Zanna, Claudia Tomasini

**Affiliations:** Dipartimento di Chimica “Giacomo Ciamician”, Università di Bologna, Via Selmi, 2, 40126 Bologna, Italy; demetra.giuri2@unibo.it (D.G.); nicola.zanna@studio.unibo.it (N.Z.)

**Keywords:** lauric acid, l-Dopa, organogel, rheology

## Abstract

We prepared the small pseudopeptide Lau-l-Dopa(OBn)_2_-d-Oxd-OBn (Lau = lauric acid; l-Dopa = l-3,4-dihydroxyphenylalanine; d-Oxd = (4*R*,5*S*)-4-methyl-5-carboxyl-oxazolidin-2-one; Bn = benzyl) through a number of coupling reactions between lauric acid, protected l-Dopa and d-Oxd with an excellent overall yield. The ability of the product to form supramolecular organogels has been tested with different organic solvents of increasing polarity and compared with the results obtained with the small pseudopeptide Fmoc-l-Dopa(OBn)_2_-d-Oxd-OBn. The mechanical and rheological properties of the organogels demonstrated solvent-dependent properties, with a storage modulus of 82 kPa for the ethanol organogel. Finally, to have a preliminary test of the organogels’ ability to adsorb pollutants, we treated a sample of the ethanol organogel with an aqueous solution of Rhodamine B (RhB) for 24 h. The water solution slowly lost its pink color, which became trapped in the organogel.

## 1. Introduction

Low-molecular-weight gelators (LMWG) are an interesting class of molecules that are able to form supramolecular structures [1,2]. These molecules, having a molecular weight lower than 1000 Da and a specific stereochemistry, can self-assemble thanks to weak interactions, including with hydrogen bonds, π-π stacking and Van der Waals forces. Gelation process starts with the dissolution of the LMWG into a solvent; by means of a trigger, gelator molecules start assembling in long structures, most commonly fibers, which entangle leading to networks that are able to immobilize the solvent [3,4,5]. 

The critical parameters for the formation of gels are the gelator concentration, the solvent properties and the trigger of the process [6,7,8]. The content of gelator in a gel is usually very small, since the percentage of solid phase can be even less than 1%. The rest of the material is constituted by liquid phase, usually a solvent that can partially dissolve the gelator. The transformation process of the solution containing the gelator in a gel is due to the addition of an external stimulus (a trigger), such as temperature variation [9,10], ultrasound sonication, pH change [11,12,13,14] or the addition of a chemical species [15,16,17,18] which enables the alignment of the fibers. 

Gel formation often requires several hours to be completed. By contrast, when ultrasound sonication is used as a trigger, gelation can occur instantly at room temperature, which makes these gels highly attractive for several applications [8]. When the gel is formed, no solvent flow is observed when inverting the test tube upside-down, although the solvent mobility is still possible on a molecular level.

LMW-gels are receiving great interest because of the possibility to tailor the gelator structure through simple synthetical steps, the ability to obtain materials with different properties and the gels being suitable for several applications [1,19,20]. However, it is still challenging to predict the gelation attitude of these molecules from their structure, as a small modification can lead to a gel having different properties, or could even prevent its formation [21]. 

Gels formed from organic solvents (organogels) have been known for decades and have been applied for several industrial applications [3,22,23]. The high solvent content of these materials may facilitate the diffusion of small molecules from another liquid. Moreover, the high surface area and porosity of the gel structure together with the possibility to form a number of non-covalent interactions may have a key role in molecules absorption. This effect may be applied to the removal of unwanted pollutants from water, such as spill oil [4,24,25,26,27], toxic ions or dyes, also considering that gels are solid-like materials from the rheological point of view, and can be handled as solids [28]. Moreover, it is possible to exploit some other properties of gels (i.e., thixotropy) in several applications, from paints and cleaning materials, to dermo-cosmetics and personal care products, to nutraceutics and food processing [20,29].

## 2. Results and Discussion

A recent study on the possibility of developing a general road map to an *ab initio* design of a gelator or a gel, has demonstrated that is not possible to achieve such a goal, as more molecular-level insights into the self-assembly process leading to gelation are required [19,30,31].

Instead, success might come through systematic studies on a particular class of molecules. As a part of this process, we are investigating derivatives of the l-Phe-d-Oxd moiety [l-Phe = l-phenylalanine; d-Oxd = (4*R*,5*S*)-4-methyl-5-carboxyl-oxazolidin-2-one]. The d-Oxd moiety introduced is a little molecule that mimics a constrained proline group and is able to block the peptide bond that is always in the *trans* conformation [32,33]. We have successfully utilized d-Oxd in the formation of several supramolecular materials [34,35,36], including hydrogels [37,38,39].

In this paper we want to investigate the gelling activity of small pseudopeptide built replacing l-Phe with a fully protected l-Dopa (l-Dopa = 3,4-dihydroxyphenylalanine) and coupled with Lau (Lau = lauric acid) [20,40], that is a long chain fatty acid, particularly suitable to form micelles, supramolecular fibers and gels [41,42,43]. The replacement of l-Phe with l-Dopa allows the introduction of two new functional groups on the gelator: we selected two Bn (benzyl) as protecting groups of the hydroxyl groups on the aromatic ring, for their possibility to form π-π stacking interactions between different gelator molecules.

**Scheme 1:**
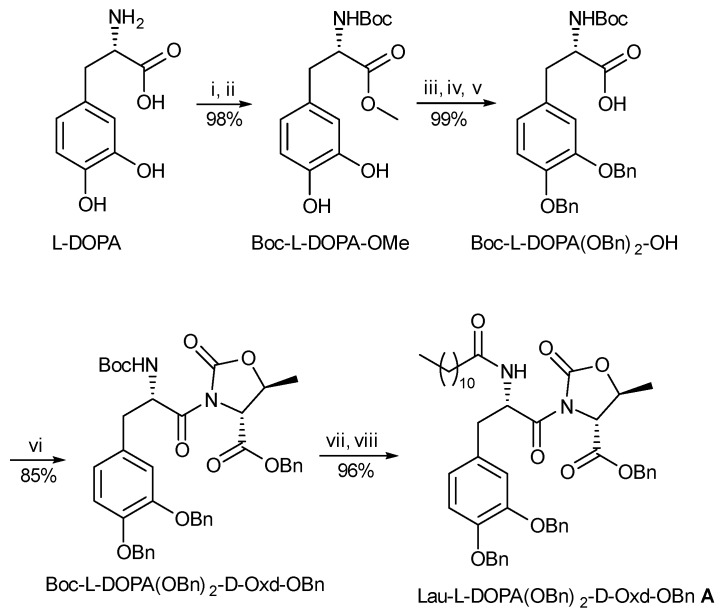
Reagents and conditions: (i) SOCl_2_ (excess), MeOH, 0 °C, 24 h; (ii) Boc_2_O (2 equiv.), NaHCO_3_ (2 equiv.), THF/H_2_O, r.t., 18 h; (iii) BnBr (2.2 equiv.), K_2_CO_3_ (2.2 equiv.), TBAB (0.2 equiv.), NaI (0.2 equiv.), acetone, reflux, 4 h; (iv) 1M NaOH, MeOH/THF, r.t., 18 h; (v) 1M HCl; (vi) D-Oxd-OBn (1 equiv.), HBTU (1.1 equiv.), DIEA (2 equiv.), dry ACN, r.t., 4 h; (vii) TFA (18 equiv.), CH_2_Cl_2_, r.t., 4 h; (viii) lauric acid (1 eq.), HBTU (1.1 eq.), DIEA (2.2 eq.), dry ACN, r.t. 2 h.

Gelator Lau-l-Dopa(OBn)_2_-d-Oxd-OBn (**A**) was prepared by modification of a procedure already reported for the preparation of Fmoc-l-Dopa(OBn)_2_-d-Oxd-OBn (Fmoc = fluorenylmethoxycarbonyl) [44]. The synthesis started from the unprotected and commercially available L-Dopa that was transformed in four steps into Boc-l-Dopa(OBn)_2_-OH (Boc = *tert*-butyloxycarbonyl group) in a multigram scale with excellent yields. After coupling with the D-Oxd moiety, the Boc group was replaced with the long chain of a fatty acid (Scheme 1). Gelator **A** was obtained pure as a white solid in 67% overall yield from l-Dopa.

**Figure 1:**
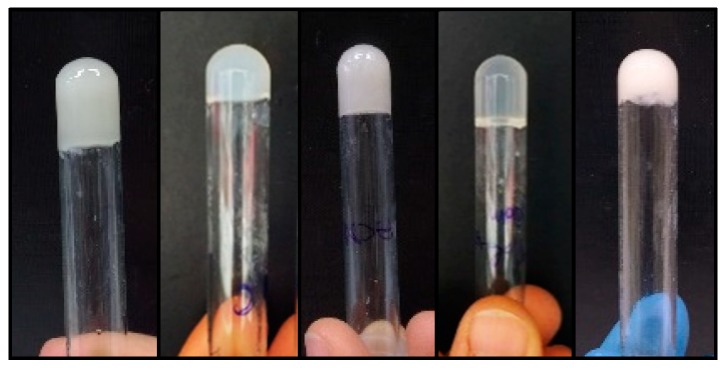
Photographs of the organogels obtained using a 2% *w*/*w* concentration of gelator **A** in the following solvents listed as function of their polarity (from left to right): toluene, ethyl acetate, acetonitrile, ethanol, methanol.

The ability of pseudopeptide **A** to form organogels was tested under several conditions. In a typical procedure, the molecule was mixed with an organic solvent in 1% and 2% *w*/*w* concentration, then the mixture was sonicated for a time ranging between 15 and 30 min to find out the best conditions to form the gel. After several attempts, the best operating conditions include 2% *w*/*w* gelator concentration and 20 min sonication, because in any case no organogel was obtained with a 1% *w*/*w* gelator concentration. The general method for the organogels preparation is to place a portion (20 mg) of gelator **A** in a test tube with 1 mL of the organic solvent. The mixture is stirred and sonicated at room temperature for 20 min, and then it is allowed to stand quiescently for 16 h. In Figure 1, we show the picture of some representative samples of organogels prepared with this technique. They all look homogeneous and opaque. Interestingly, the previously reported Fmoc-l-Dopa(OBn)_2_-d-Oxd-OBn [44] never forms organogels under any conditions, either using organic solvents or solvents mixtures. This effect may be due to the tendency of the saturated long chain of lauric acid to stick together producing fibers or micelles [45]. The formation of hydrogels failed because gelator **A** is insoluble in water, even if the mixture is sonicated for 30 min.

In Table 1 we report the results obtained preparing organogels under these conditions as a function of different solvents, that have been listed as a function of increasing polarity, using a scale where water is 100 [46], from very apolar toluene to very polar methanol. In any case, an organogel is formed. 

The first test to measure organogels properties is the dropping ball test that enables us to measure the organogel melting point. The melting points obtained with toluene, ethyl acetate and acetonitrile range all between 40 and 50 °C. In contrast, a thermally stronger gel was obtained with the polar ethanol, while methanol produces a very weak organogel, probably due to a limited solubility of gelator A in the solvent. The solvents boiling points (also listed in Table 1) do not appear to be strictly correlated to the organogels melting points.

**Figure 2:**
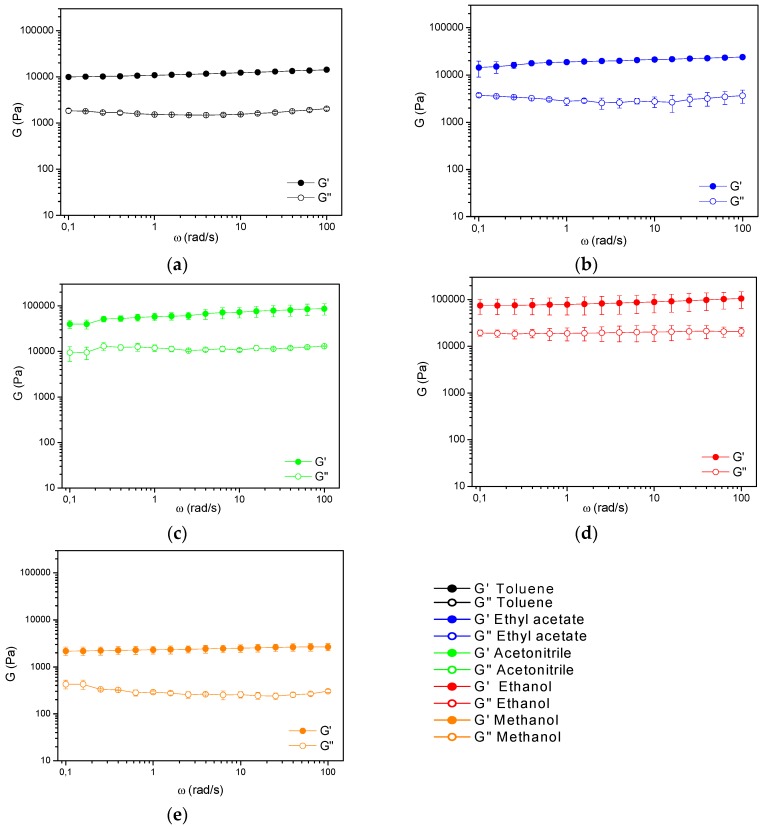
Frequency sweep experiments performed on the 2% *w*/*w* gels in the solvents listed by increasing polarity: (**a**) toluene (black); (**b**) ethyl acetate (blue); (**c**) acetonitrile (green); (**d**) ethanol (red); (**e**) methanol (orange). The analyses were performed on the gels about 20 hours after the gelation begun. For more details see the Materials and Methods section.

Good rheological properties are usually associated with a higher T_gel_ [14], so we measured the viscoelastic behavior of the five organogels listed in Table 1: the storage and loss moduli (G′ and G″ respectively) were evaluated by amplitude sweep (Figure S1) and frequency sweep analysis at 23 °C (Figure 2). All the organogels tested are characterized by a “solid-like” behavior, i.e., the storage modulus is approximately an order of magnitude higher than the loss component. In Table 1 we reported the average G′ and G″ values recorded from the frequency sweep experiments (γ = 0.04%) of the organogels (ω = 1 s^−1^). As we had foreseen, the organogels strength nicely correlates with the melting points measurements, confirming that ethanol provides the gel with the strongest network. The measures have been all repeated (the mean measurements and the standard deviation bars are shown in Figure 2).

The analysis of the rheological properties of ethanol organogel was repeated at different gelator concentrations (1.5% and 3% *w*/*w* concentration). From the comparison of the frequency sweep experiments (Figure S2), we can notice that the G′ modulus deeply increases, moving from 1.5% to the 2% *w*/*w* concentration. In contrast, no change in the frequency dependent behavior is detected, if the organogel concentration is further increased to 3% *w*/*w*.

Thixotropy [47,48,49,50] is a gel property related to the sol/gel equilibrium and describes the system ability to recover the gel status after a strong stress that induces transformation into sol. Moreover, self-healing property [18,48,51,52,53] may be defined as the ability to autonomously reconstruct the bonding interactions after damage. 

**Figure 3:**
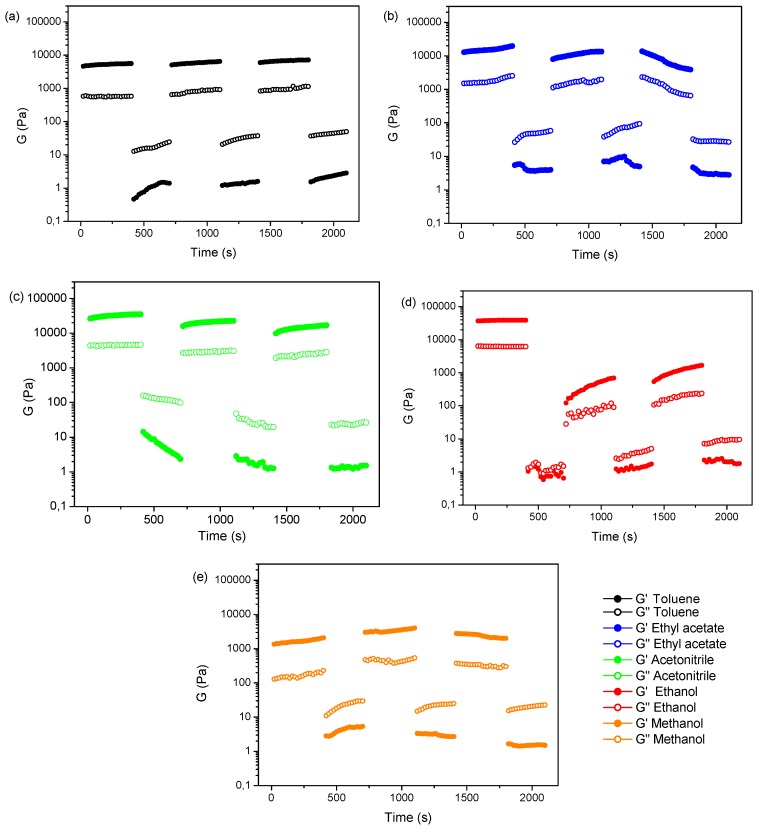
Values of storage moduli G′ (solid circles) and loss moduli G″ (empty cyrcles) recorded during step strain experiments performed on organogels in the solvent listed by increasing polarity: (**a**) toluene; (**b**) ethyl acetate; (**c**) acetonitrile; (**d**) ethanol; (**e**) methanol.

In Figure 3, we report the results obtained from measurements of the thixotropic properties of our organogels. Multiple cycles of three steps were applied to the gels. During the first step, the sample was subjected to a strain value within the LVE (linear viscoelastic) region and was characterized by G′ values greater than G″. When the applied strain was increased above the crossover point, the sample behavior switched from gel-like to sol-like, with G″ values greater than G′. Finally, the sample was left at fixed strain within the LVE range to check the recovery of the gel-like behavior. Unfortunately, the ethanol organogel has poor thixotropic properties, as its strength is highly reduced when it recovers after the strain above the crossover point. The same inconvenient does not occur for the other organogels that promptly recover their properties.

Finally, to have a preliminary test of the organogels ability to absorb pollutants [54,55], we used Rhodamine B (RhB) as model molecule. RhB, a model refractory organic dye pollutant which contains four *N*-ethyl groups at either side of the xanthene ring, was chosen as the target pollutant, being an important representative of xanthene dye that is widely used as a colorant in textiles and food stuffs, and also a well-known water tracer fluorescent [56].

**Figure 4:**
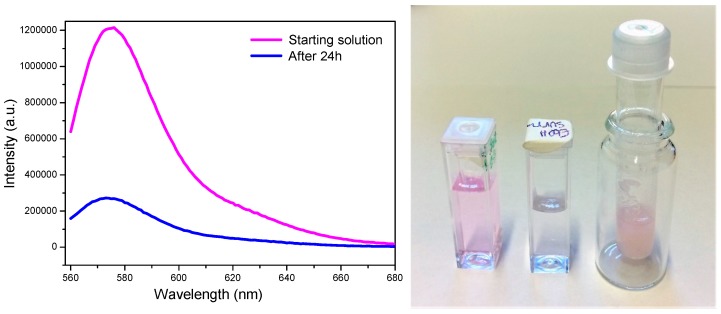
(**Left**) Emission spectra of an aqueous solution of RhB (2.0 µM) (pink) and of the same solution after being treated with the ethanol organogel for 24 hours (blue). (**Right**) Photograph of a fresh sample of the aqueous solution of RhB, of the same sample after treatment with the ethanol organogel, and of the organogel after 24 h (from left to right).

We treated a sample of the ethanol organogel with 1 mL of an aqueous solution of RhB (2 µM) for 24 h. The water solution did not mix with the gel and slowly lost its pink color that moved to the gel (Figure 4, Figures S3 and S4). To confirm this result, we measured the emission spectrum of the water solution after treatment with the ethanol organogel in comparison with a fresh sample of the aqueous solution of RhB. Under these experimental conditions, fluorescence intensity is proportional to the fluorophore concentration [57,58]. After 24 h of absorption, the spectrum intensity (and so **RhB** concentration in water) is reduced to 23% of the original sample, thus confirming that the molecule is trapped into the organogel. The analysis was repeated twice after 48 h and after 72 h, but no variation of the spectrum intensity of the sample was detected.

We also measured the amount of ethanol found in the aqueous phase after incubation by ^1^H NMR analysis (Figure S5). After calculation of the mols of water and ethanol as a function of the corresponding peak intensities, we found out that the water/ethanol ratio after 24 h is 9.5:0.5. No variations occur if the measure is repeated after 48 h and 72 h.

## 3. Conclusions

This comparative study shows the formation of organogels based on aliphatic organic solvents, as ethanol or acetonitrile. The preparation of organogels is usually based on aromatic solvents [4,19,25], or alcohol/water mixtures [24].

We have demonstrated that the pseudopeptide Lau-l-Dopa(OBn)_2_-d-Oxd-OBn is an efficient organogelator in 2% *w*/*w* concentration, as it can gelate solvents of increasing polarity, such as toluene, ethyl acetate, acetonitrile, ethanol and methanol, in contrast with Fmoc-l-Dopa(OBn)_2_-d-Oxd-OBn that never forms organogel under any conditions. The mechanical and rheological properties of the organogels demonstrated solvent-dependent properties, with a storage modulus of 82 kPa for the ethanol organogel. 

This result suggests that the saturated long chain of the lauric acid is crucial for the formation of the three-dimensional network. A sample of ethanol organogel was also treated with an aqueous solution of RhB, to have a preliminary test of its ability to adsorb pollutants. After 24 h, the emission spectrum intensity of the RhB solution is reduced to 23% of the original sample, which was not modified after 48 h and 72 h. Organogels prepared with gelators **A** may be employed in molecules absorption, to remove unwanted pollutants from water.

## 4. Materials and Methods

All chemicals and solvents were purchased from Sigma-Aldrich (St. Louis, MO, USA), VWR International (Radnor, PA, USA) or Iris Biotech GmbH (Marktredwitz, Germany) and used as received. Acetonitrile was distilled under an inert atmosphere before use. MilliQ water (Millipore, resistivity = 18.2 mΩ cm) was used throughout. All reactions were carried out in dried glassware. The melting points of the compounds are uncorrected. High quality infrared spectra (64 scans) were obtained with an ATR-FT-IR Bruker Alpha System spectrometer (64 scans). All compounds were dried in vacuo and all the sample preparations were performed in a nitrogen atmosphere. NMR spectra were recorded with a Varian Inova 400 spectrometer at 400 MHz (^1^H NMR) and 100 MHz (^13^C NMR). Chemical shifts are reported in δ values relative to the solvent peak.

### 4.1. Synthesis of Lau-l-Dopa(OBn)_2_-d-Oxd-OBn 

Boc-l-Dopa(OBn)_2_-d-Oxd-OBn was synthetized following a multistep procedure in solution and deprotected from the *N*-Boc group as reported in literature [44]. The resulting compound (1 eq.) was dissolved in a solution of dry ACN and DIEA (2.2 eq.) and added dropwise to a stirred solution of lauric acid (1 eq.) and HBTU (1.1 eq.) in dry ACN. After 2 h at r.t., the solution was evaporated under reduced pressure, the residue was dissolved in DCM and extracted with H_2_O, HCl 1M, NaHCO_3_, H_2_O. The organic layer was dried over sodium sulfate, filtered, and concentrated under reduced pressure. The residue was washed with MeOH, sonicated and filtered. The product (Lau-l-Dopa(OBn)_2_-d-Oxd-OBn) was obtained as a white solid with a yield of 85%. M.p. = 172–175 °C; IR: 3324, 1792, 1739, 1711, 1645 cm^−1^; ^1^H-NMR (CDCl_3_): δ 0.9 (t, 3H, CH_3_ Lau), 1.25 (m, 18H, CH_2_ Lau), 1.36 (d, J = 6.4 Hz, 3H, CH_3_ Oxd), 2.1 (m, 2H, CH_2_CO Lau), 2.93 (dd, J = 6.8, 13.6 Hz, 1H, C*H*Hβ-Dopa), 3.04 (dd, J = 6.0, 13.6 Hz, 1H, CH*H*β-Dopa), 4.23 (d, J = 3.9 Hz, 1H, CHN Oxd), 4.49 (dq, J = 3.9, 6.4 Hz, 1H, CHO Oxd), 5.09 (s, 2H, CH_2_Ph), 5.11 (s, 2H, CH_2_Ph), 5.18 (s, 2H, CH_2_Ph), 5.92 (d, J =7.6 Hz, 1H, NH), 6.00 (dt, J = 6.8, 7.6 Hz, 1H, CHα-Dopa), 6.69 (d, J = 8.0 Hz, 1H, CH Ar Dopa), 6.79 (s, 1H, CH Ar Dopa), 6.81 (d, J = 8.0 Hz, 1H, CH Ar Dopa), 7.24–7.45 (m, 15H, ArH); ^13^C-NMR (CDCl_3_): δ 14.1, 21.1, 22.7, 25.5, 29.2, 29.3, 29.4, 29.5, 29.6, 29.7, 31.9, 36.4, 38.1, 52.5, 61.8, 68.0, 71.3, 73.6, 115.0, 116.3, 122.3, 127.2, 127.4, 127.7, 127.8, 128.4, 128.5, 128.7, 128.8, 128.9, 134.6, 137.2, 137.3, 148.2, 148.9, 151.0, 167.3, 172.2.

### 4.2. Conditions for the Gel Formation

All organogels were prepared in a concentration of 2% *w*/*w* under the following conditions. 20 mg of gelator (Lau-l-Dopa(OBn)_2_-d-Oxd-OBn) were placed in a test tube (8 mm of diameter) with 1 mL of organic solvent (see Table 1). The solution was stirred for 5 min and sonicated for 20 min in order to allow the complete dissolution of the sample and the formation of the organogel. All samples were allowed to stand quiescently for 16 hours before further characterization.

### 4.3. Conditions for T_gel_ Determination

T_gel_ can be considered as the melting temperature of a gel. It was determined by placing a small glass ball (diameter: 5 mm, weight: 165 mg) inside the test tube (8 mm of diameter) containing the gel. When the gel is correctly formed, the ball is suspended on the top of it. The test tube is then gradually heated (2 °C/min): T_gel_ is the temperature in which the ball penetrates inside the gel, because the network is dissolved. Some gel samples melt, producing a clear solution (and this was the behavior of our organogels), while in other cases the gelator shrinks and the solvent is ejected, as syneresis occurs. 

### 4.4. Rheology

Rheology experiments were carried out on an Anton Paar Rheometer MCR 102 using a parallel plate configuration (25 mm diameter). Experiments were performed at constant temperature of 23 °C controlled by the integrated Peltier system. All the analysis (amplitude sweep, frequency sweep and thixotropy) were performed with fixed gap value of 0.5 mm. The samples were prepared the day before the analysis and left overnight at controlled temperature of 20 °C to complete the gelation process. Oscillatory amplitude sweep experiments (γ: 0.01−100%) were carried out at fixed frequency of 10 rad·s^−1^, in order to determine the linear viscoelastic (LVE) range and the crossover point of the gels (G″ > G′). Once established the LVE of each organogel, frequency sweep tests were performed (ω: 0.1–100 rad·s^−1^) at constant strain within the LVE region of each sample (γ = 0.04%). To verify the thixotropic properties of the samples, strain values within the crossover point region (for 400 s) and over the crossover point region (for 300 s) were consecutively applied to the organogels, for three cycles. The values of the applied strain were selected on the basis of the crossover point value obtained from amplitude sweep experiment (within the crossover point: γ = 0.04%, ω = 10 s^−1^; over the crossover point: γ = 100%, ω = 10 s^−1^).

### 4.5. Fluorescence Spectroscopy

The organogels were prepared the night before the measurement in a concentration of 2% w/w under the aforementioned conditions. 1 mL of aqueous solution of Rhodamine B (2.0 µM) was then placed on the top of the EtOH gel and left in the dark for 24 h (ratio gelator/Rhodamine = 12.9 × 10^3^). The above solution was analyzed in disposable cuvettes with optical path length of 1.0 cm. Fluorescence spectra were collected with a Fluoromax-4 spectrofluorometer Horiba Jobin Yvon (emission spectra: λ_exc_ = 550 nm; λ_em_: 560–700 nm). 

## Figures and Tables

**Table 1:** Summary of the conditions for organogel formation using gelators **A** in different solvents (all measurements were done in triplicate).

Solvent	Solvent Polarity ^1^	Solvent b.p. (°C)	Organogel m.p. (°C)	G′ (KPa)	G″ (KPa)
Toluene	9.9	110.6	40–41	10	1.5
Ethyl acetate	23	77	40–43	19	2.9
Acetonitrile	46	81.6	42–45	56	12.2
Ethanol	65.4	78.5	60–65	82	19
Methanol	76.2	64.6	25–28	2.3	0.3

^1^ Water is 100 [46].

## Supplementary Materials

The following are available online at https://www.mdpi.com/2310-2861/5/2/27/s1.

## References

[1] Sangeetha N.M., Maitra U. (2005). Supramolecular gels: Functions and uses. Chem. Soc. Rev..

[2] Yu G., Yan X., Han C., Huang F. (2013). Characterization of supramolecular gels. Chem. Soc. Rev..

[3] Draper E.R., Adams D.J. (2017). Low-Molecular-Weight Gels: The State of the Art. Chem.

[4] Haldar D., Podder D., Roy Chowdhury S., Nandi S. (2019). Tripeptide based super-organogelators: Structure and function. New J. Chem..

[5] Pramanik A., Paikar A., Haldar D. (2015). Sonication-induced instant fibrillation and fluorescent labeling of tripeptide fibers. RSC Adv..

[6] Xiao S., Zou Y., Yu M., Yi T., Zhou Y., Li F., Huang C. (2007). A photochromic fluorescent switch in an organogel system with non-destructive readout ability. Chem. Commun..

[7] Du X., Zhou J., Shi J., Xu B. (2015). Supramolecular Hydrogelators and Hydrogels: From Soft Matter to Molecular Biomaterials. Chem. Rev..

[8] Yu X., Chen L., Zhang M., Yi T. (2014). Low-molecular-mass gels responding to ultrasound and mechanical stress: Towards self-healing materials. Chem. Soc. Rev..

[9] Overstreet D.J., Dutta D., Stabenfeldt S.E., Vernon B.L. (2012). Injectable hydrogels. J. Polym. Sci. Part B Polym. Phys..

[10] Bhattacharjee S., Maiti B., Bhattacharya S. (2016). First report of charge-transfer induced heat-set hydrogel. Structural insights and remarkable properties. Nanoscale.

[11] Zhou S.L., Matsumoto S., Tian H.D., Yamane H., Ojida A., Kiyonaka S., Hamachi I. (2005). pH-responsive shrinkage/swelling of a supramolecular hydrogel composed of two small amphiphilic molecules. Chem. A Eur. J..

[12] Adams D.J., Mullen L.M., Berta M., Chen L., Frith W.J. (2010). Relationship between molecular structure, gelation behaviour and gel properties of Fmoc-dipeptides. Soft Matter.

[13] Sutton S., Campbell N.L., Cooper A.I., Kirkland M., Frith W.J., Adams D.J. (2009). Controlled release from modified amino acid hydrogels governed by molecular size or network dynamics. Langmuir.

[14] Zanna N., Merlettini A., Tatulli G., Milli L., Focarete M.L., Tomasini C. (2015). Hydrogelation induced by Fmoc-protected peptidomimetics. Langmuir.

[15] Zanna N., Focaroli S., Merlettini A., Gentilucci L., Teti G., Falconi M., Tomasini C. (2017). Thixotropic Peptide-Based Physical Hydrogels Applied to Three-Dimensional Cell Culture. ACS Omega.

[16] Zheng Y.J., Loh X.J. (2016). Natural rheological modifiers for personal care. Polym. Adv. Technol..

[17] Chakraborty P., Gazit E. (2018). Amino Acid Based Self-assembled Nanostructures: Complex Structures from Remarkably Simple Building Blocks. Chem. Nano. Mat..

[18] Zanna N., Merlettini A., Tomasini C. (2016). Self-healing hydrogels triggered by amino acids. Org. Chem. Front..

[19] Dastidar P. (2008). Supramolecular gelling agents: Can they be designed?. Chem. Soc. Rev..

[20] Uzan S., Barış D., Çolak M., Aydın H., Hoşgören H. (2016). Organogels as novel carriers for dermal and topical drug delivery vehicles. Tetrahedron.

[21] Van Esch J.H. (2009). We can design molecular gelators, but do we understand them?. Langmuir.

[22] Esposito C.L., Kirilov P., Roullin V.G. (2018). Organogels, promising drug delivery systems: An update of state-of-the-art and recent applications. J. Control. Release.

[23] Ajayaghosh A., Praveen V.K., Vijayakumar C., Sommerdijk N.A.J.M., Ajayaghosh A., Meskers S.C.J., Schenning A.P.H.J., Silva C., Friend R.H., Aida T. (2008). Organogels as scaffolds for excitation energy transfer and light harvesting. Chem. Soc. Rev..

[24] Bera S., Haldar D. (2016). A rechargeable self-healing safety fuel gel. J. Mater. Chem. A.

[25] Vibhute A.M., Muvvala V., Sureshan K.M. (2016). A Sugar-Based Gelator for Marine Oil-Spill Recovery. Angew. Chem. Int. Ed..

[26] Li J., Huo Y., Zeng H. (2018). Combinatorial identification of a highly soluble phase-selective organogelator with high gelling capacity for crude oil gelation. J. Mater. Chem. A.

[27] Ren C., Chen F., Zhou F., Shen J., Su H., Zeng H. (2016). Low-Cost Phase-Selective Organogelators for Rapid Gelation of Crude Oils at Room Temperature. Langmuir.

[28] Okesola B.O., Smith D.K. (2016). Applying low-molecular weight supramolecular gelators in an environmental setting-self-assembled gels as smart materials for pollutant removal. Chem. Soc. Rev..

[29] Mohanty S., Wu Y., Chakraborty N., Mohanty P., Ghosh G. (2016). Impact of alginate concentration on the viability, cryostorage, and angiogenic activity of encapsulated fibroblasts. Mater. Sci. Eng. C.

[30] Dastidar P. (2019). Designing Supramolecular Gelators: Challenges, Frustrations, and Hopes. Gels.

[31] Lan Y., Corradini M.G., Weiss R.G., Raghavan S.R., Rogers M.A. (2015). To gel or not to gel: Correlating molecular gelation with solvent parameters. Chem. Soc. Rev..

[32] Lucarini S., Tomasini C. (2001). Synthesis of oligomers of trans-(4S,5R)-4-carboxybenzyl 5-methyl oxazolidin-2-one: An approach to new foldamers. J. Org. Chem..

[33] Tomasini C., Trigari V., Lucarini S., Bernardi F., Garavelli M., Peggion C., Formaggio F., Toniolo C. (2003). Pseudopeptide foldamers—The homo-oligomers of benzyl (4S,5R)-5-methyl-2-oxo-1,3-oxazolidine-4-carboxylate. Eur. J. Org. Chem..

[34] Angelici G., Castellucci N., Falini G., Huster D., Monari M., Tomasini C. (2010). Pseudopeptides designed to form supramolecular helixes: The role of the stereogenic centers. Cryst. Growth Des..

[35] Angelici G., Falini G., Hofmann H.J., Huster D., Monari M., Tomasini C. (2009). Nanofibers from oxazolidi-2-one containing hybrid foldamers: What is the right molecular size?. Chem.—A Eur. J..

[36] Angelici G., Castellucci N., Contaldi S., Falini G., Hofmann H.J., Monari M., Tomasini C. (2010). A network of small molecules connected by cross-linked NH bonds. Cryst. Growth Des..

[37] Castellucci N., Sartor G., Calonghi N., Parolin C., Falini G., Tomasini C. (2013). A peptidic hydrogel that may behave as a “trojan Horse”. Beilstein J. Org. Chem..

[38] Castellucci N., Angelici G., Falini G., Monari M., Tomasini C. (2011). L-Phe-D-Oxd: A privileged scaffold for the formation of supramolecular materials. Eur. J. Org. Chem..

[39] Castellucci N., Falini G., Angelici G., Tomasini C. (2011). Formation of gels in the presence of metal ions. Amino Acids.

[40] Cardoso A.Z., Mears L.L.E., Cattoz B.N., Griffiths P.C., Schweins R., Adams D.J. (2016). Linking micellar structures to hydrogelation for salt-triggered dipeptide gelators. Soft Matter.

[41] Streck L., Caroni A.L.P.F., Fonseca J.L.C. (2018). Temperature and composition effects on the morphology of o/w dispersions based on poly(oxyethylene 20) sorbitan monolaurate and sorbitan monolaurate. Colloids Surf. A Physicochem. Eng. Asp..

[42] Galindo-Alvarez J., Sadtler V., Marchal P., Perrin P., Tribet C., Marie E., Durand A. (2012). Nanoemulsions with enhanced temperature stability using thermo-sensitive association of nonionic surfactant and amphiphilic polyelectrolytes. Colloids Surf. A Physicochem. Eng. Asp..

[43] Shah D.O., Ranch K.M., Desai A.R., Choksi H.H., Vyas B.A., Patil R.J., Maulvi F.A. (2017). Effect of surfactant chain length on drug release kinetics from microemulsion-laden contact lenses. Int. J. Pharm..

[44] Zanna N., Iaculli D., Tomasini C. (2017). The effect of L-DOPA hydroxyl groups on the formation of supramolecular hydrogels. Org. Biomol. Chem..

[45] Latxague L., Gaubert A., Maleville D., Baillet J., Ramin M.A., Barthélémy P. (2016). Carbamate based bolaamphiphile as Low Molecular Weight Hydrogelators. Gels.

[46] Smallwood I.M. (2015). Handbook of organic solvents properties. Oppor. Emerg. Mark..

[47] Gong Z., Yang Y., Ren Q., Chen X., Shao Z. (2012). Injectable thixotropic hydrogel comprising regenerated silk fibroin and hydroxypropylcellulose. Soft Matter.

[48] Liu Y., Ling S., Wang S., Chen X., Shao Z. (2014). Thixotropic silk nanofibril-based hydrogel with extracellular matrix-like structure. Biomater. Sci..

[49] Pek Y.S., Wan A.C.A., Ying J.Y. (2010). The effect of matrix stiffness on mesenchymal stem cell differentiation in a 3D thixotropic gel. Biomaterials.

[50] Li Y., Zhou F., Wen Y., Liu K., Chen L., Mao Y., Yang S., Yi T. (2014). (-)-Menthol based thixotropic hydrogel and its application as a universal antibacterial carrier. Soft Matter.

[51] Basak S., Nanda J., Banerjee A. (2014). Multi-stimuli responsive self-healing metallo-hydrogels: Tuning of the gel recovery property. Chem. Commun..

[52] Karan C.K., Bhattacharjee M. (2016). Self-Healing and Moldable Metallogels as the Recyclable Materials for Selective Dye Adsorption and Separation. ACS Appl. Mater. Interfaces.

[53] Roy S., Baral A., Banerjee A. (2013). An amino-acid-based self-healing hydrogel: Modulation of the self-healing properties by incorporating carbon-based nanomaterials. Chem. Eur. J..

[54] Jeon Y.S., Lei J., Kim J.H. (2008). Dye adsorption characteristics of alginate/polyaspartate hydrogels. J. Ind. Eng. Chem..

[55] Naskar J., Palui G., Banerjee A. (2009). Tetrapeptide-based hydrogels: For encapsulation and slow release of an anticancer drug at physiological ph. J. Phys. Chem. B.

[56] Wang W., Chen X., Hong C., Zhu F., Yao Y., Xue Z. (2012). Oxidation Degradation of Rhodamine B in Aqueous by UV/S2O8 2− Treatment System. Int. J. Photoenergy.

[57] Montalti M., Credi A., Prodi L., Gandolfi M.T. (2006). Handbook of Photochemistry.

[58] Genovese D., Bonacchi S., Juris R., Montalti M., Prodi L., Rampazzo E., Zaccheroni N. (2013). Prevention of Self-Quenching in Fluorescent Silica Nanoparticles by Efficient Energy Transfer. Angew. Chem. Int. Ed..

