# Supplementary material for: Low Molecular Weight Gelators Based on Functionalized l-Dopa Promote Organogels Formation
Source: Gels. 2019 May 14;5(2):27. doi: 10.3390/gels5020027 (PMC6630615; doi:10.3390/gels5020027)
Supplement: Supplementary file 1 [file gels-05-00027-s001.pdf]

Supporting Information

Low Molecular Weight Gelators Based on Functionalized L-Dopa Promote Organogels Formation

Demetra Giuri [1], Nicola Zanna [1], Claudia Tomasini [1*]

[1] Dipartimento di Chimica "Giacomo Ciamician" - Università di Bologna - Via Selmi, 2 – 40126 Bologna - Italy

* Correspondence: claudia.tomasini@unibo.it; Tel.: +39-0512099486

Contents

**Figure S1.** Amplitude Sweep experiments	page S2
**Figure S2.** Comparison between different concentrations of organogelator in EtOH	page S3
**Figure S3.** RhB emission in water and in water/ethanol solution	page S4
**Figure S4.** RhB lost during the emission experiment	page S5
**Figure S5.** ^{1}H-NMR quantification of the water/ethanol mixture in RhB absorption experiment	page S6-S7

Amplitude Sweep experiments

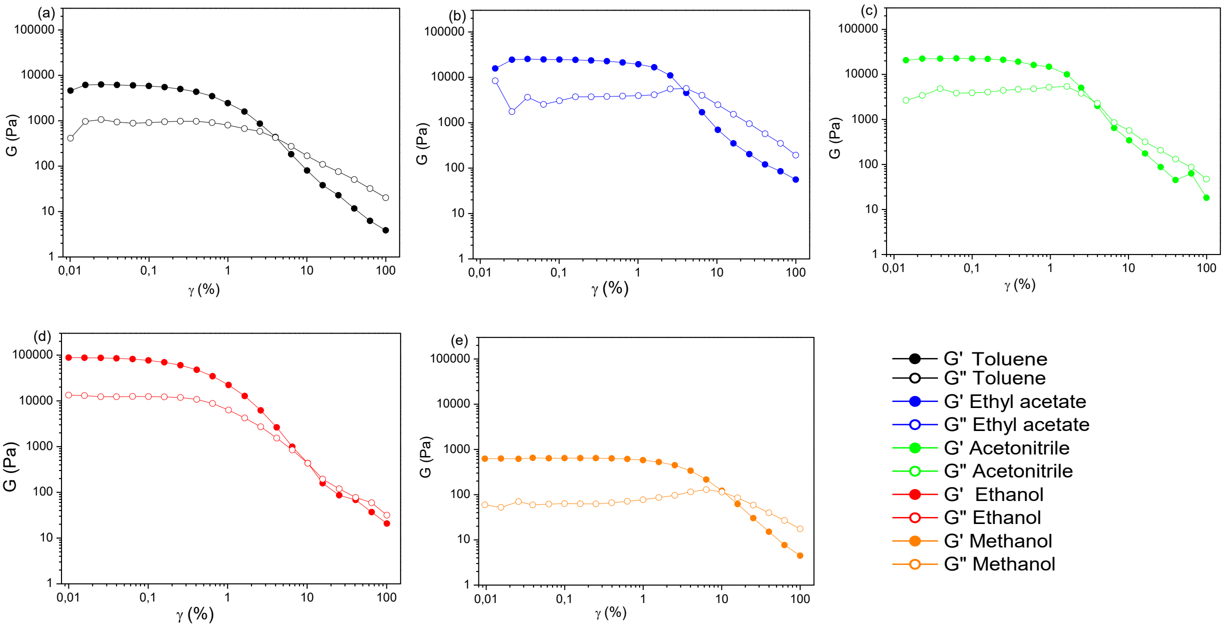

Figure S1. Amplitude Sweep experiments of the organogels, made with the 2% w/w gelator concentration of gelator (A) in the solvents listed by increasing polarity: (a) toluene (black); (b) ethyl acetate (blue); (c) acetonitrile (green); (d) ethanol (red); (e) methanol (orange). The analyses were performed on the gels about 20 hours after the gelation begun. (Storage modulus (solid circles) and loss modulus (empty circles)).

Comparison between different gelator concentrations

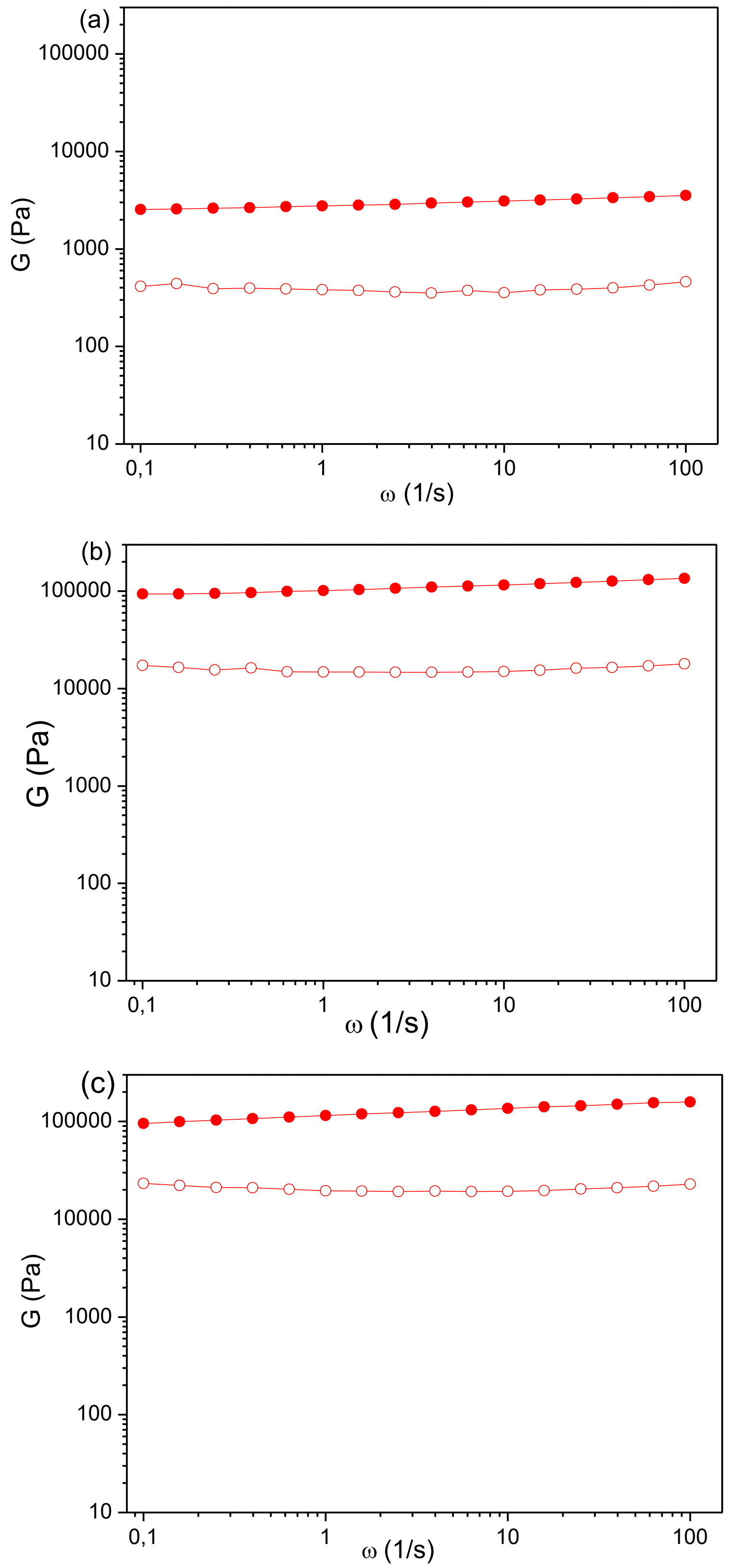

Figure S2. Frequency Sweep experiments of an ethanol organogel 1.5% concentration w/w (a), 2% concentration w/w (b) and 3% concentration w/w (c).

RhB emission in water and in water/ethanol solution

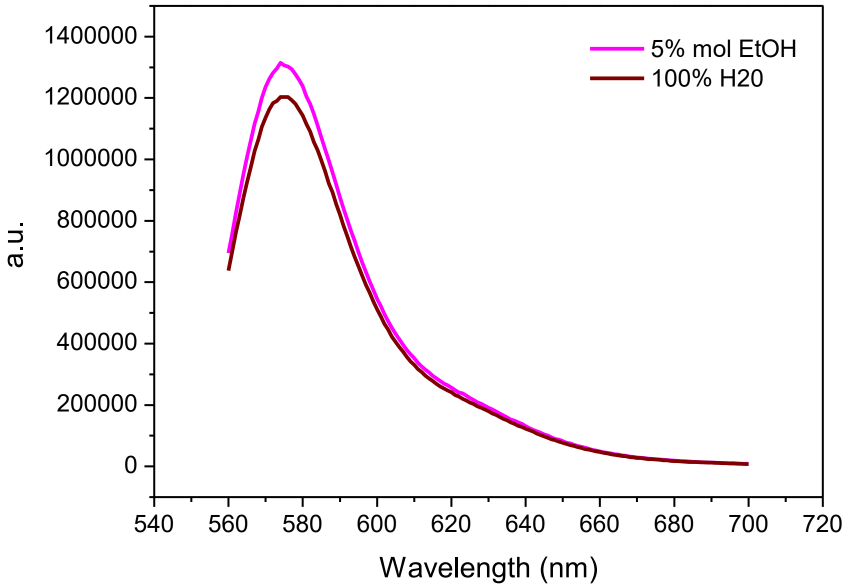

Figure S3. Comparison of the emission spectra of the RhB solution in water (wine) and in water/ethanol (5% molar) solution (pink).

RhB lost during the emission experiment

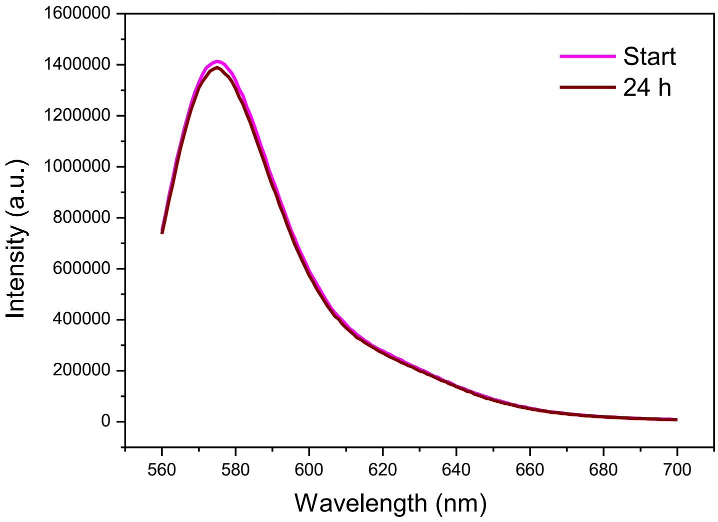

Figure S4. Emission Spectra of the starting solution of RhB in water (pink). This was left for 24 h in the same test tube used for making the gels in the absorption experiment, then transferred again in a cuvette for checking the emission (wine), demonstrating that RhB is not lost during the experiment (e.g. no absorption to walls).

^{1}H-NMR quantification of the water/ethanol mixture in RhB absorption experiment

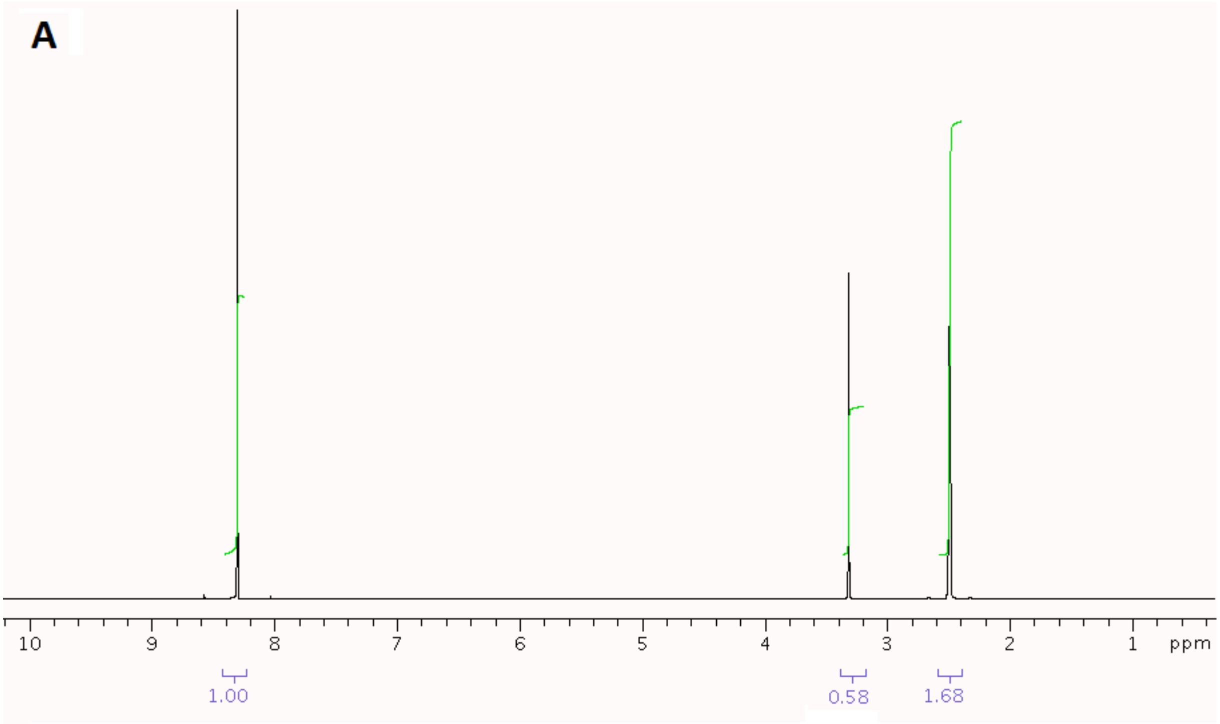

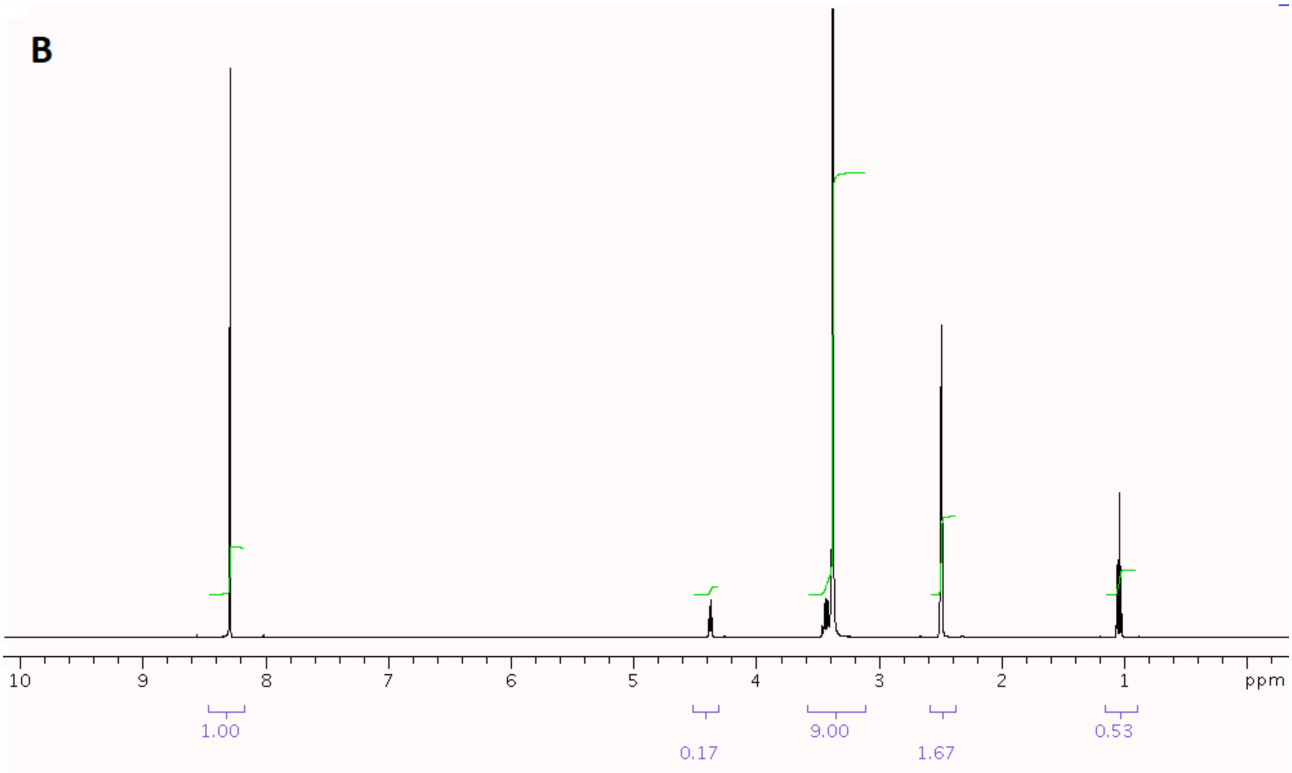

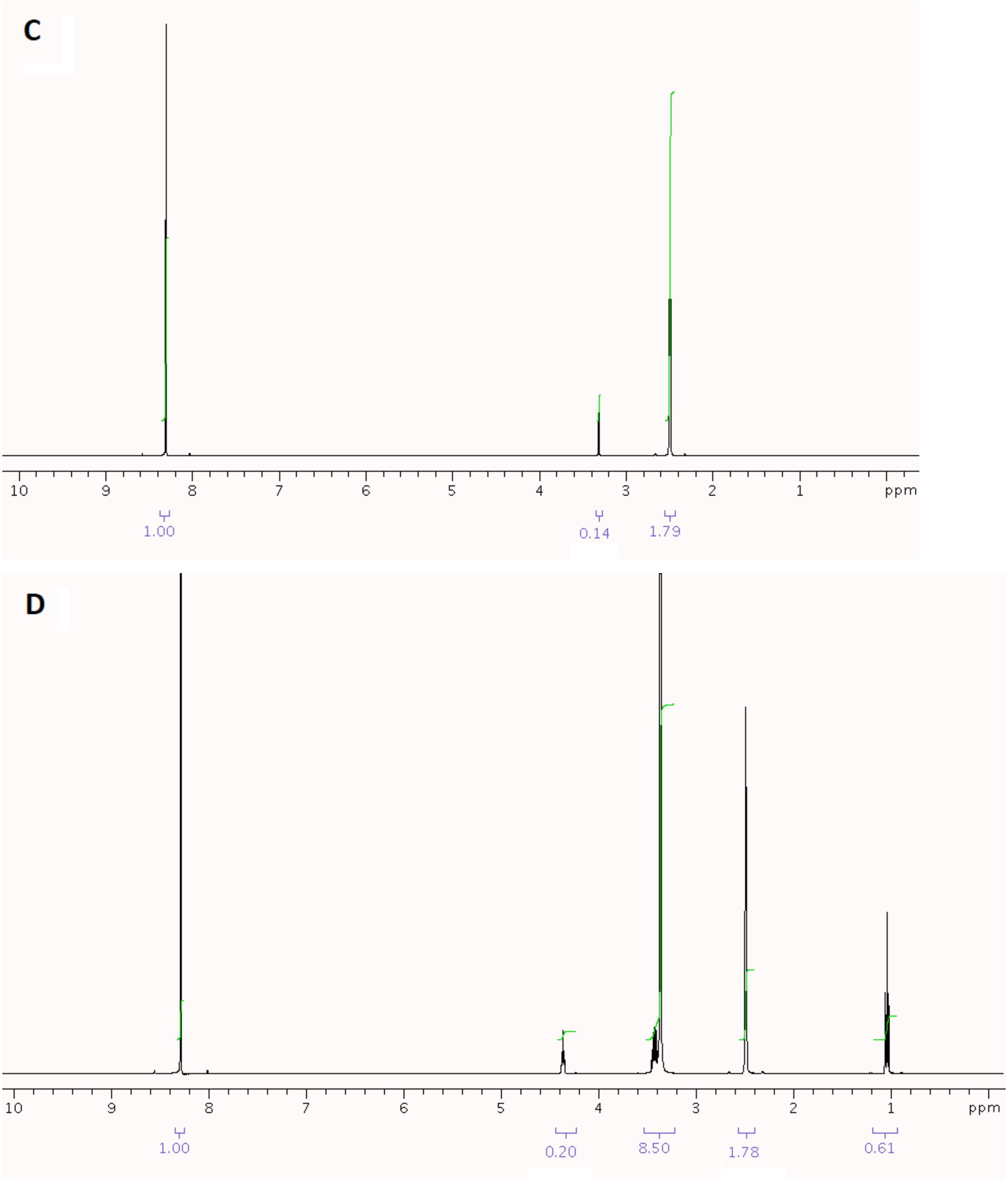

Figure S5. ^{1}H-NMR spectra of the starting solution $CHCl_3$ (as internal standard) in DMSO (A) and the same solution after the addition of 10 μL of the Water/Ethanol solution, 24h after the absorption of RhB (B); spectra of the starting solution $CHCl_3$ (as internal standard) in DMSO (C) and the same solution after the addition of 10 μL of the Water/Ethanol solution, 48h after the absorption of RhB (D).
